# Aqueous Extract of *Bacopa procumbens* and the NAPEL Formulation Mitigate MPTP-Induced Neurotoxicity via Nrf2/HSF1/HIF-1α Signaling in a Parkinson’s Disease Model

**DOI:** 10.3390/ijms262411914

**Published:** 2025-12-10

**Authors:** Maribel Pérez-Rodríguez, Salvador Pérez-Mora, Marvin A. Soriano-Ursúa, María del Consuelo Gómez-García, Yazmin Montserrat Flores-Martinez, Juan Ocampo-López, Absalom Zamorano-Carrillo, José Manuel Viveros-Bartolomé, David Guillermo Pérez-Ishiwara

**Affiliations:** 1 Laboratorio de Biomedicina Molecular I, Escuela Nacional de Medicina y Homeopatía (ENMyH), Instituto Politécnico Nacional, Mexico City 07320, Mexico; mperezr2103@alumno.ipn.mx (M.P.-R.); sperezm1510@alumno.ipn.mx (S.P.-M.); cgomezg@ipn.mx (M.d.C.G.-G.); yfloresma@ipn.mx (Y.M.F.-M.); jviverosb2000@alumno.ipn.mx (J.M.V.-B.); 2 Departamento de Investigación, Atria Scientific, Av. de los Maestros 452, Nueva Santa María, Azcapotzalco, Mexico City 02800, Mexico; 3 Laboratorio de Neurofisiología, Escuela Superior de Medicina (ESM), Instituto Politécnico Nacional, Plan de San Luis y Diaz Mirón s/n, Mexico City 11340, Mexico; msoriano@ipn.mx; 4 Laboratorio de Histología e Histopatología, Área Académica de Medicina Veterinaria, Instituto de Ciencias Agropecuarias (ICAp), Universidad Autónoma del Estado de Hidalgo, Tulancingo de Bravo, Hidalgo 43600, Mexico; jocampo@uaeh.edu.mx; 5 Laboratorio Biofísica Computacional, Escuela Nacional de Medicina y Homeopatía (ENMyH), Instituto Politécnico Nacional, Mexico City 07320, Mexico; azamorano@ipn.mx

**Keywords:** *Bacopa procumbens*, NAPEL, Parkinson’s disease, MPTP, neuroprotection, oxidative stress, Keap1/Nrf2/ARE pathway, HSF1, HIF-1α, antioxidant enzymes, lipid peroxidation, interactomic analysis, functional enrichment analysis

## Abstract

Parkinson’s disease (PD) is a progressive neurodegenerative disorder characterized by dopaminergic neuron degeneration in the substantia nigra and striatum. Current treatments are largely palliative and frequently associated with adverse effects. This study aimed to evaluate the neuroprotective potential of an aqueous extract of *Bacopa procumbens* (*B. procumbens*) and the NAPEL formulation—composed of five neuroactive compounds (**N**aringenin, **A**pigenin, **P**aeoniflorin, (−)-**E**picatechin, and **L**upeol)—in a murine model of MPTP-induced parkinsonism. Behavioral, histological, and molecular parameters were examined to elucidate underlying mechanisms of neuroprotection. Male mice received MPTP to induce parkinsonism, followed by oral administration of *B. procumbens* extract or NAPEL. Motor function was assessed through open-field-related parameters. Substantia nigra neuronal morphology was analyzed histologically. Molecular analyses focused on the Keap1/Nrf2/ARE pathway, HSF1, HIF-1α, antioxidant enzymes, and lipid peroxidation. Additionally, in silico analyses (GeneMANIA, STRING) were performed to explore regulatory networks associated with Nrf2, HSF1, and HIF-1α. The aqueous extract significantly improved motor performance, increased rearing events, enhanced central exploration, and increased total distance traveled. It preserved neuronal number and soma diameter in the substantia nigra. Molecularly, the extract activated the Keap1/Nrf2/ARE axis and induced HSF1 and HIF-1α, accompanied by increased SOD-1, CAT, and GSR expression and reduced lipid peroxidation. NAPEL also produced behavioral and histological improvements but did not activate Nrf2, HSF1, or HIF-1α nor notably elevate antioxidant enzymes, except for CAT in the striatum. In silico analyses identified Nrf2, HSF1, and HIF-1α as central nodes integrating oxidative stress, proteostasis, hypoxia, inflammation, and apoptotic responses. These findings support the neuroprotective potential of both *B. procumbens* aqueous extract and the NAPEL formulation, highlighting their value as promising therapeutic candidates for Parkinson’s disease.

## 1. Introduction

Parkinson’s disease (PD) is the second most common neurodegenerative disorder worldwide, affecting over 10 million people, with projections estimating ~25.2 million cases by 2050 [1,2]. Clinically, PD manifests as bradykinesia, resting tremor, rigidity, and postural instability, while pathologically it is characterized by the progressive loss of dopaminergic neurons in the substantia nigra and the accumulation of α-synuclein-containing Lewy bodies [3]. PD arises from a multifactorial interplay of genetic susceptibility and environmental factors, such as pesticides, neurotoxins, certain drugs, and infections [4,5]. Key pathogenic processes include oxidative stress, mitochondrial dysfunction, and neuroinflammation [6]. Experimentally, the neurotoxin MPTP is widely used in C57BL/6 mice because it reproduces hallmark features of PD. After crossing the blood–brain barrier, MPTP is converted by astrocytic MAO-B into MPP^+^, which accumulates in dopaminergic neurons via the dopamine transporter and inhibits mitochondrial complex I, triggering energy failure, oxidative stress, and inflammation, central events in Parkinsonian neurodegeneration [7,8].

Current treatments such as L-DOPA and other pharmacological therapies alleviate motor symptoms but do not halt or reverse neurodegeneration [9,10]. This limitation has increased interest in therapeutic strategies targeting oxidative stress [11,12]. Among these, the Keap1/Nrf2/ARE pathway is a central endogenous defense system, regulating antioxidant enzymes—including HO-1, SOD-1, CAT, and GSR—through activation of the transcription factor Nrf2 [13], which has been shown to protect dopaminergic neurons in MPTP models [14].

Other stress-responsive transcription factors, such as hypoxia-inducible factor 1-alpha (HIF-1α) [15] and heat shock factor 1 (HSF1) [16] have emerged as key regulators of the neuroprotective response. HIF-1α and HSF1 are transcription factors that orchestrate cellular responses to stress [17]. HIF-1α activates genes involved in angiogenesis, metabolism, mitochondrial regulation, and antioxidant defense [18,19,20], while HSF1 induces molecular chaperones that preserve proteostasis [21,22,23]. In PD, HIF-1α promotes dopaminergic neuron survival by reducing oxidative stress and mitochondrial toxicity [24], whereas HSF1 prevents α-synuclein misfolding and aggregation [25].

Several plant-derived natural compounds have demonstrated neuroprotective effects in experimental models of PD, particularly through activation of the Keap1/Nrf2/ARE pathway [11,12]. For example, ginkgolide A from *Ginkgo biloba* [26], curcumin from *Curcuma longa* [27], baicalein from *Scutellaria baicalensis* [28], and rosmarinic acid from *Rosmarinus officinalis* [29] have each been shown to modulate this pathway favorably in MPTP-induced models, reducing oxidative damage and preserving motor function.

Currently, the combined contribution of Nrf2, HSF1, and HIF-1α has not been examined in PD, nor has their potential integrated modulation of shared molecular targets and biological processes been mapped. This represents a relevant gap, as these pathways converge on mechanisms central to dopaminergic vulnerability.

Our research group has characterized the chemical composition of the aqueous extract of *B. procumbens* and demonstrated both its antioxidant activity and its wound-healing properties [30,31], and hair-growth-promoting effects [32,33]. However, neither the aqueous extract of *B. procumbens* nor its metabolites had been evaluated in PD. Furthermore, the mechanisms underlying NAPEL—an optimized formulation composed of five neuroactive metabolites (**N**aringenin, **A**pigenin, **P**aeoniflorin, (−)-**E**picatechin, and **L**upeol), whose name derives from the initials of these compounds—also remain unknown.

Considering these gaps, our study provides a comparative evaluation of both treatments in the MPTP model and assesses their capacity to modulate integrated cytoprotective pathways. Additionally, through in silico analyses, we mapped the potential molecular targets and biological processes regulated by Nrf2, HSF1, and HIF-1α. By constructing an interactomic network, we identified shared nodes that may underlie the neuroprotective effects observed. Overall, this study addresses a critical gap and offers new mechanistic insights into plant-derived neuroprotection in PD.

## 2. Results

### 2.1. Motor Function Tests

**Figure 1:**
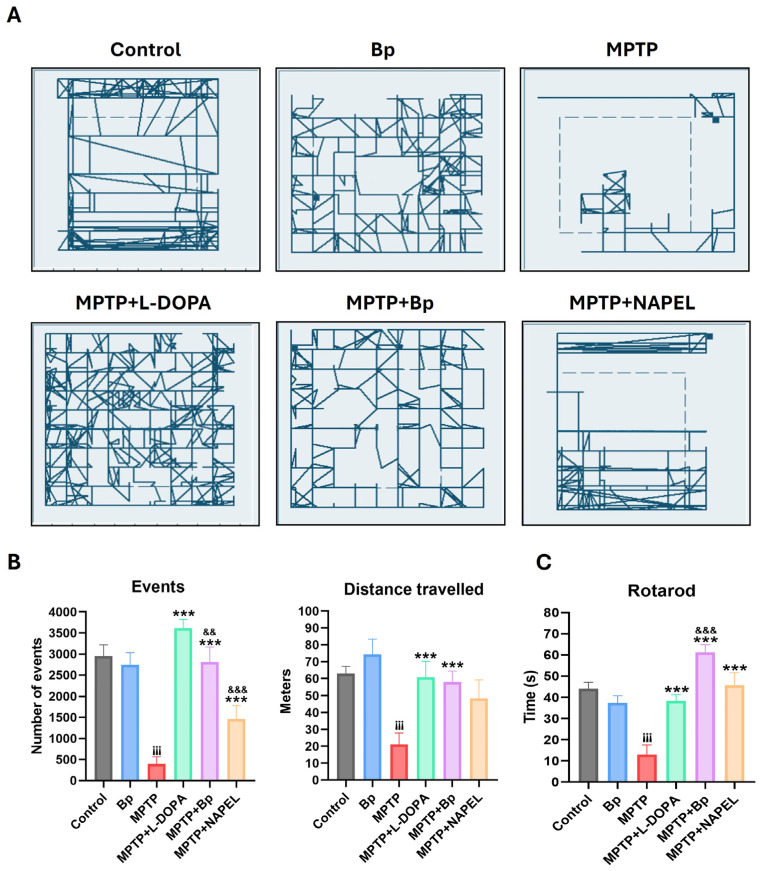
Motor evaluation in mice with MPTP-induced parkinsonism. (**A**) Open field trajectories (5 min) of each group. (**B**) Total number of events and total distance traveled. (**C**) Latency to fall in the rotarod test. Data are expressed as mean ± standard deviation (*n* = 6 per group). Two-way ANOVA followed by Tukey’s post hoc test was applied. For all symbols (¡, *, &), statistical significance was defined as follows: two symbols = *p* ≤ 0.002; three symbols = *p* ≤ 0.001. Different symbols were used for comparisons: “¡” for control vs. MPTP, “*” for MPTP vs. treated groups, and “&” for MPTP+L-DOPA vs. MPTP+Bp and MPTP+NAPEL. Bp; extract of *B. procumbens*, L-DOPA; levodopa.

In the open field test (Figure 1A), control mice exhibited active displacements, with trajectories concentrated mainly along the peripheral zones and, to a lesser extent, in the central region of the arena, covering a total distance of 62.8 m. The Bp group traveled a distance of 74.4 m and displayed a more homogeneous and evenly distributed exploratory pattern throughout the arena, suggesting stable locomotion under physiological conditions.

Regarding the number of events recorded in the open field test (Figure 1B), understood as the number of movement detections registered by the tracking software sensors (indirect indicators of exploratory activity), both the control and Bp groups exhibited high mean values, 2948 and 2744 events, respectively, reflecting robust exploratory behavior. In contrast, the MPTP group showed a dramatic reduction, recording only 399 events. The MPTP+L-DOPA and MPTP+NAPEL groups reached intermediate values, 3616 and 1460, respectively, whereas the MPTP+Bp group displayed an almost complete recovery, with 2812 events, a number comparable to the control and Bp groups. This finding suggests a significant neuroprotective effect of Bp treatment on exploratory behavior.

When assessing locomotor activity, the MPTP group exhibited a marked reduction, covering only 21.1 m, the lowest distance among all experimental groups, with exploration restricted to the periphery of the arena. The MPTP+L-DOPA group showed the highest displacement (60.7 m), with a broad and evenly distributed exploration pattern. Similarly, the MPTP+Bp group reached a distance of 57.8 m, with a displacement pattern comparable to that of the Bp group, suggesting functional recovery of locomotion. In contrast, the MPTP+NAPEL group showed a slightly lower displacement (55.3 m) than MPTP+Bp, with more limited exploration predominantly in the peripheral areas.

In the rotarod motor performance test (Figure 1C), the MPTP group showed a significant impairment, with an average latency to fall of approximately 13 s, compared with the control (44 s) and Bp (37.3 s) groups. The MPTP+L-DOPA group exhibited improved performance, with an average of 38.3 s, whereas the MPTP+Bp group achieved the longest time recorded (61.2 s), even surpassing the L-DOPA-treated group. The MPTP+NAPEL group reached an intermediate latency of approximately 45.6 s.

### 2.2. Histological Evaluation of the Substantia Nigra by Luxol Fast Blue Staining

**Figure 2:**
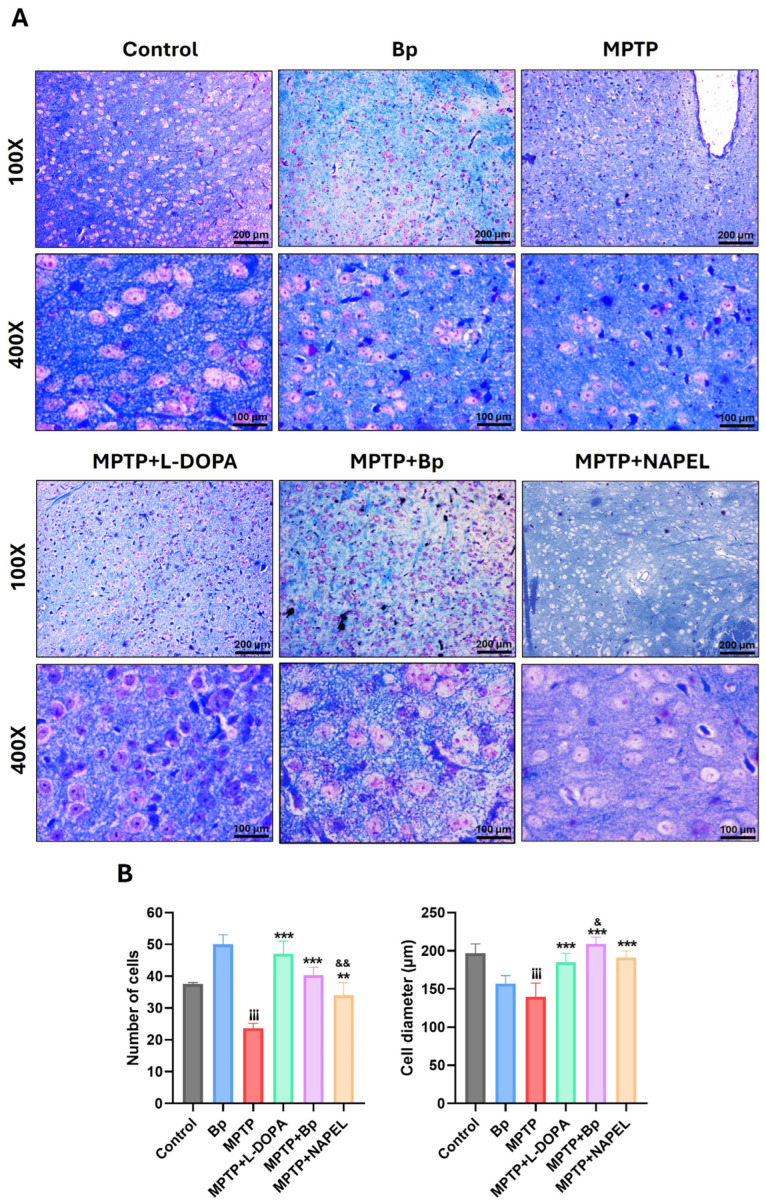
Histological evaluation of the substantia nigra by Luxol Fast Blue staining. (**A**) Representative microphotographs of each group. (**B**) Quantification of the average number and diameter (µm) of cells per field (400×). Data are expressed as mean ± standard deviation (*n* = 3 per group). Two-way ANOVA followed by Tukey’s post hoc test was applied. For all symbols (¡, *, &), statistical significance was defined as follows: one symbol = *p* ≤ 0.033; two symbols = *p* ≤ 0.002; three symbols = *p* ≤ 0.001. Different symbols were used for comparisons: “¡” for control vs. MPTP, “*” for MPTP vs. treated groups, and “&” for MPTP+L-DOPA vs. MPTP+Bp and MPTP+NAPEL. Bp; extract of *B. procumbens*, L-DOPA; levodopa. Scale bars = 200 and 100 µm.

Luxol Fast Blue staining was used to evaluate cellular integrity in the substantia nigra across the different experimental groups (Figure 2), as this region is the main site of MPTP-induced dopaminergic degeneration and more accurately reflects neuronal damage in PD models [34].

At 400× magnification, control mice showed an average of 38 cells per field, whereas the Bp group exhibited a significant increase, reaching 50 cells. As expected, the MPTP group displayed a marked reduction in cell density, averaging 24 cells. Interestingly, the MPTP+L-DOPA group demonstrated substantial recovery, with 47 cells, even surpassing the control group. The MPTP+Bp and MPTP+NAPEL groups presented intermediate values of 40 and 34 cells per field, respectively.

Regarding the mean cell diameter in the same fields, the control and Bp groups showed values of 196.7 and 156.9 µm, respectively. In the MPTP group, a significant reduction was observed, with an average of 139.6 µm, reflecting structural damage. In contrast, the MPTP+L-DOPA (184.8 µm), MPTP+Bp (208.9 µm), and MPTP+NAPEL (191.3 µm) groups showed diameters comparable to or even greater than those of the control group, suggesting a protective or restorative effect against MPTP-induced damage.

### 2.3. Modulation of the Endogenous Antioxidant System

**Figure 3:**
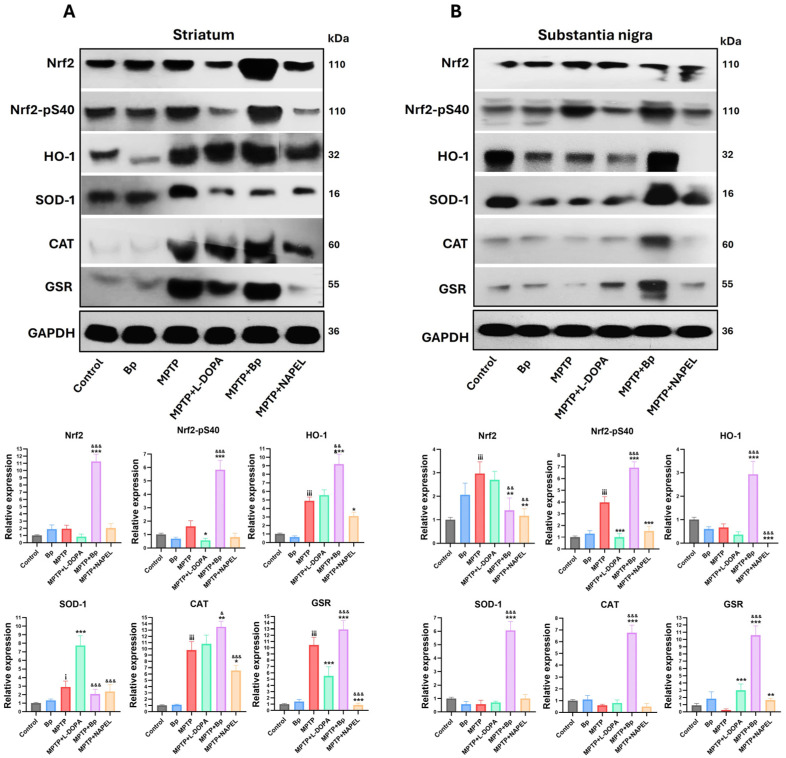
Relative expression of antioxidant proteins by Western blot. (**A**) Expression of Nrf2, Nrf2-pSer40 (levels), HO-1, SOD1, CAT, and GSR in the striatum (**B**) and in the substantia nigra. Relative protein expression was normalized to GAPDH. Data are expressed as mean ± standard deviation (*n* = 3 per group). Two-way ANOVA followed by Tukey’s post hoc test was applied. For all symbols (¡, *, &), statistical significance was defined as follows: one symbol = *p* ≤ 0.033; two symbols = *p* ≤ 0.002; three symbols = *p* ≤ 0.001. Different symbols were used for comparisons: “¡” for control vs. MPTP, “*” for MPTP vs. treated groups, and “&” for MPTP+L-DOPA vs. MPTP+Bp and MPTP+NAPEL. Bp; extract of *B. procumbens*, L-DOPA; levodopa.

In the striatum (Figure 3A and Supplementary Table S1), the control and Bp groups did not show significant changes in the expression of Nrf2, p-Nrf2, HO-1, SOD-1, CAT, and GSR. However, MPTP administration induced a significant increase in the antioxidant enzymes HO-1, SOD-1, CAT, and GSR, indicating a compensatory activation of the antioxidant system in response to neurotoxic damage. In the MPTP+L-DOPA group, decreased levels of Nrf2-pS40 and reduced GSR expression were observed, along with an increase in SOD1 compared with the MPTP group. On the other hand, treatment with MPTP+NAPEL markedly reduced the expression of HO-1, CAT, and GSR relative to the MPTP group. Notably, the MPTP+Bp group exhibited the greatest activation of the antioxidant system: Nrf2 increased 10.43-fold, Nrf2-pS40 5.27-fold, HO-1 4.30-fold, CAT 3.70-fold, and GSR 2.53-fold, whereas SOD-1 showed a slight decrease (0.83-fold) compared with MPTP. These results indicate that the Bp extract was more effective in enhancing the antioxidant pathway than either the NAPEL formulation or L-DOPA.

In the substantia nigra (Figure 3B and Supplementary Table S1), the control and Bp groups did not show relevant variations in the expression of the evaluated proteins. In contrast, MPTP administration induced a marked increase in total Nrf2 expression and in the levels of Nrf2-pS40. In the MPTP+L-DOPA group, GSR expression was increased while Nrf2-pS40 levels decreased compared with MPTP. The MPTP+NAPEL group exhibited a reduction in all proteins analyzed, with the exception of SOD-1 and CAT, which did not show significant changes relative to MPTP. Remarkably, the MPTP+Bp group also promoted the greatest activation of the antioxidant system in this region, with increases in Nrf2-pS40 (2.97-fold), HO-1 (2.26-fold), SOD-1 (5.50-fold), CAT (6.17-fold), and GSR (10.33-fold) compared with MPTP. This effect surpasses both the NAPEL formulation and L-DOPA treatment in positively regulating the antioxidant pathway.

**Figure 4:**
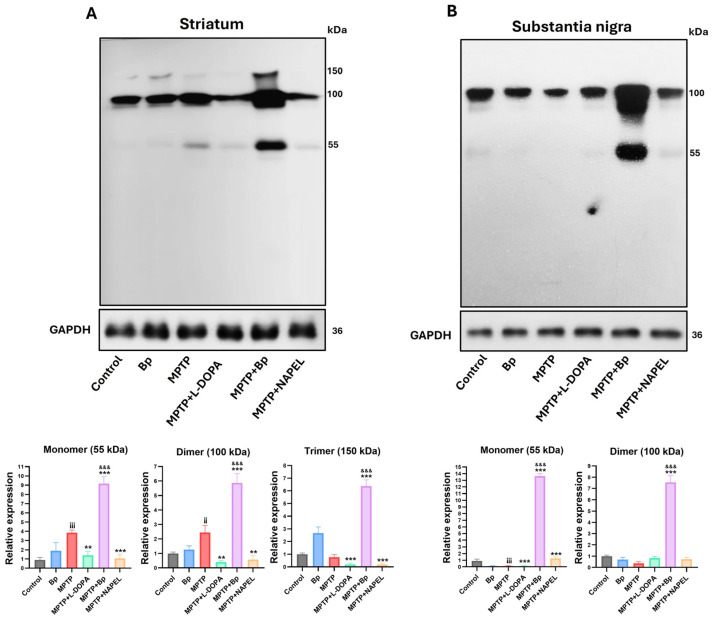
Relative expression of HSF1 conformations by Western blot. (**A**) Relative expression of HSF1 in the striatum and (**B**) in the substantia nigra. Relative protein expression was normalized to GAPDH. Data are expressed as mean ± standard deviation (*n* = 3 per group). Two-way ANOVA followed by Tukey’s post hoc test was applied. For all symbols (¡, *, &), statistical significance was defined as follows: two symbols = *p* ≤ 0.002; three symbols = *p* ≤ 0.001. Different symbols were used for comparisons: “¡” for control vs. MPTP, “*” for MPTP vs. treated groups, and “&” for MPTP+L-DOPA vs. MPTP+Bp and MPTP+NAPEL. Bp; extract of *B. procumbens*, L-DOPA; levodopa.

In addition to evaluating proteins associated with the antioxidant pathway, the expression of HSF1 was analyzed due to its role in the modulation of oxidative stress [35]. In the striatum, the three conformations of HSF1 were identified: monomer (~55 kDa), dimer (~100 kDa), and trimer (~150 kDa) (Figure 4A and Supplementary Table S2). In the control group and in animals treated only with the Bp extract, these conformations were present at basal levels, with the dimer showing the highest expression. Following MPTP administration, an increase in the monomeric and dimeric conformations was observed, whereas the trimer remained similar to the control group. In the MPTP+L-DOPA and MPTP+NAPEL groups, all three conformations decreased relative to MPTP. In contrast, treatment with MPTP+Bp significantly increased the three conformations, with 5.3-fold, 3.44-fold, and 5.60-fold increases in the monomer, dimer, and trimer, respectively, compared with MPTP, indicating an active activation or restoration of HSF1 function in response to cellular damage.

In the substantia nigra, HSF1 expression exhibited a differential pattern compared with that observed in the striatum (Figure 4B and Supplementary Table S2). Across all experimental groups, only the monomeric and dimeric conformations were detected, with no clear evidence of the trimeric form. In the control group, both conformations were expressed at basal levels. In the MPTP+L-DOPA group, a reduction in the monomer and a slight increase in the dimer were observed compared with MPTP. The MPTP+NAPEL group showed an increase in the monomeric form, while the dimer remained unchanged. Remarkably, treatment with MPTP+Bp induced a robust increase in both conformations, with a 13.50-fold elevation in the monomer and a 7.16-fold increase in the dimer compared with MPTP. These results further support that the metabolites contained in the aqueous extract of Bp exert a more effective effect on HSF1 activation in both brain regions evaluated, compared with L-DOPA and the NAPEL formulation.

### 2.4. Neuroprotection Against Lipid Peroxidation

**Figure 5:**
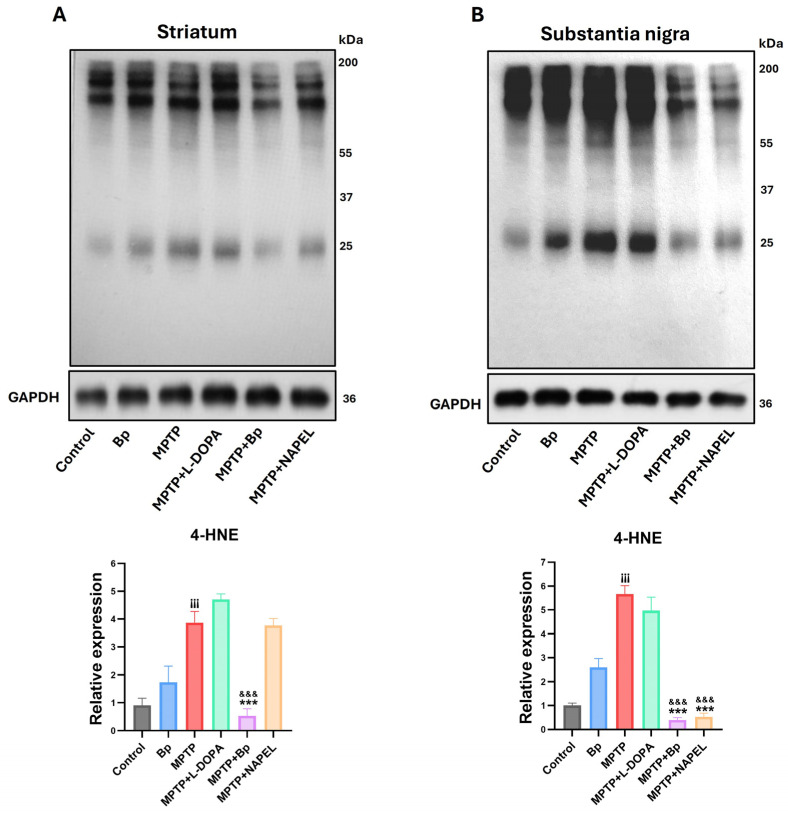
Evaluation of the lipid peroxidation marker 4-HNE by Western blot. (**A**) Detection of 4-HNE levels in the striatum and (**B**) in the substantia nigra. Quantification was performed considering the total signal per lane, since 4-HNE forms adducts with multiple proteins, generating a series of bands distributed along the gel. The total intensity of all bands detected by the antibody was summed and normalized to GAPDH. Data are expressed as mean ± standard deviation (*n* = 3 per group). Two-way ANOVA followed by Tukey’s post hoc test was applied. Three symbols indicate *p* ≤ 0.001. Different symbols were used for comparisons: “¡” for control vs. MPTP, “*” for MPTP vs. treated groups, and “&” for MPTP+L-DOPA vs. MPTP+Bp and MPTP+NAPEL. Bp; extract of *B. procumbens*, L-DOPA; levodopa.

Lipid peroxidation was assessed using the marker 4-hydroxynonenal (4-HNE) in the striatum (Figure 5A and Supplementary Table S3). Basal levels were observed in the control group, which remained unchanged in the group treated only with the aqueous extract of *B. procumbens*. In contrast, the MPTP group showed a significant increase in 4-HNE levels compared with control, indicating enhanced oxidative damage. In the MPTP+L-DOPA and MPTP+NAPEL groups, the levels of lipid peroxidation remained essentially unchanged relative to MPTP. Notably, the MPTP+Bp group exhibited a 3.34-fold decrease in 4-HNE levels compared with MPTP, demonstrating a protective effect of the *B. procumbens* extract against neurotoxin-induced lipid peroxidation.

In the substantia nigra (Figure 5B and Supplementary Table S3), a pattern similar to that observed in the striatum was found regarding the detection of the lipid peroxidation marker 4-HNE. Levels of 4-HNE remained within basal ranges in both the control group and the group treated only with the aqueous extract of *B. procumbens*, with no significant differences between them. In the MPTP+L-DOPA group, a slight reduction was observed, whereas the MPTP+Bp and MPTP+NAPEL groups showed significant decreases in 4-HNE levels compared with MPTP, by 5.27- and 5.14-fold, respectively.

### 2.5. Expression of Proteins Associated with Cell Signaling and Stress Adaptation

**Figure 6:**
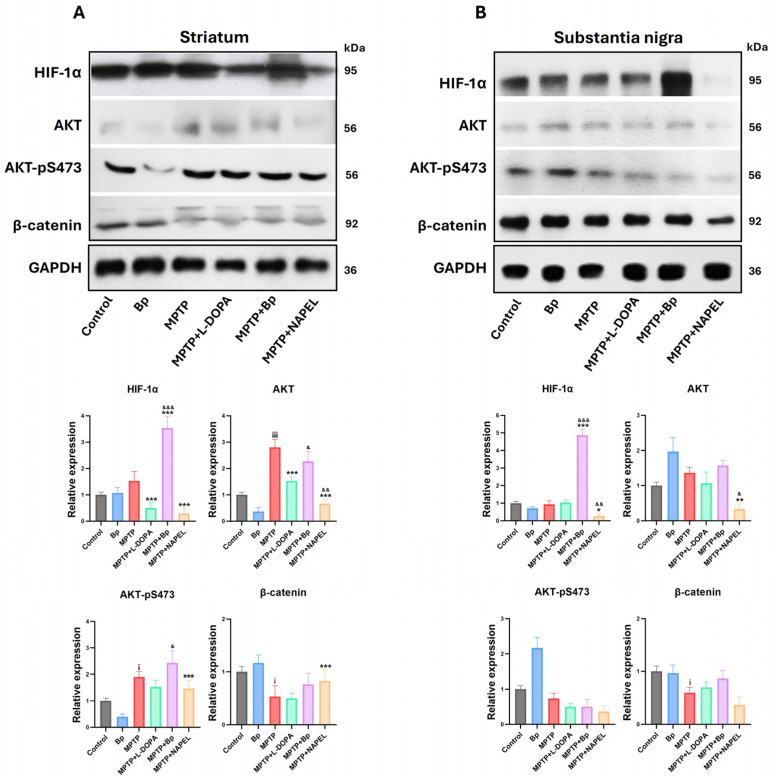
Relative expression of proteins associated with cell signaling and stress adaptation. (**A**) Protein expression of HIF-1α, AKT, AKT-pS473, and β-catenin in the striatum and (**B**) in the substantia nigra. Relative protein expression was normalized to GAPDH. Data are expressed as mean ± standard deviation (*n* = 3 per group). Two-way ANOVA followed by Tukey’s post hoc test was applied. For all symbols (¡, *, &), statistical significance was defined as follows: one symbol = *p* ≤ 0.033; two symbols = *p* ≤ 0.002; three symbols = *p* ≤ 0.001. Different symbols were used for comparisons: “¡” for control vs. MPTP, “*” for MPTP vs. treated groups, and “&” for MPTP+L-DOPA vs. MPTP+Bp and MPTP+NAPEL. Bp; extract of *B. procumbens*, L-DOPA; levodopa.

In the striatum, the expressions of HIF-1α, AKT, AKT-pS473, and β-catenin remained at basal levels in the control group, while in the group treated with the aqueous extract of *B. procumbens* slight changes were observed that were not statistically significant. Following MPTP administration, both total AKT expression and AKT-pS473 levels increased, while β-catenin expression was significantly reduced. In contrast, HIF-1α did not show significant differences relative to the control group. In the MPTP+L-DOPA group, a decrease in HIF-1α and AKT expression was detected, while AKT-pS473 and β-catenin remained unchanged compared with MPTP. In the MPTP+NAPEL group, both HIF-1α and AKT decreased, whereas AKT-pS473 and β-catenin showed no relevant modifications. In contrast, treatment with MPTP+Bp induced a significant increase in HIF-1α, with a 2.00-fold elevation compared with the MPTP group, without detectable changes in the other proteins (Figure 6A and Supplementary Table S4).

In the substantia nigra, the expression of the four proteins evaluated remained at basal levels in the control group and showed no significant changes in the group treated only with the aqueous extract of *B. procumbens*. Remarkably, in the MPTP+Bp group a significant increase in HIF-1α expression was observed, with a 3.94-fold elevation compared with MPTP. By contrast, in the MPTP+NAPEL group a significant decrease in HIF-1α and AKT expression was detected. In the remaining experimental groups, no relevant changes were observed in the expression of the analyzed proteins compared with MPTP (Figure 6B and Supplementary Table S4).

### 2.6. Nrf2, HSF1, and HIF-1α as Central Nodes of Neuroprotection

**Figure 7:**
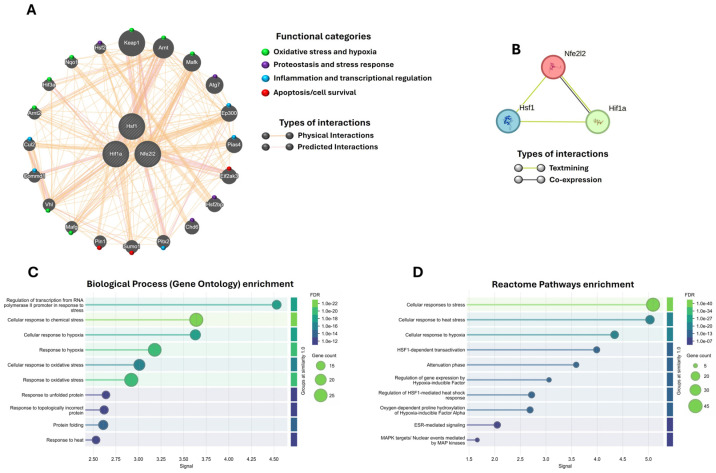
Interactome and functional enrichment analysis of Nrf2 (Nfe2l2), HSF1, and HIF-1α. (**A**) Gene interaction network generated with GeneMANIA. Genes were grouped into functional categories using different colors (circles). (**B**) Protein–protein interaction network obtained with STRING. (**C**) Gene Ontology enrichment analysis (biological processes) and (**D**) Reactome enrichment analysis for the 100 proteins with the highest probability of association (≥70%). The enrichment results show only the top 10 pathways in each analysis. The False Discovery Rate (FDR) is represented by a color scale (greener shades indicate higher significance), and circle size reflects the number of genes associated with each pathway. A stronger signal along the X-axis indicates greater enrichment, signifying a higher representation of associated genes compared to random expectations.

Using GeneMANIA, we integrated a joint gene interaction network of *Nrf2* (*Nfe2l2*), *Hsf1*, and *Hif-1α*, simulating their behavior under our experimental conditions. In *M. musculus*, the 20 most associated genes were identified (Figure 7A). The analysis revealed that these three transcription factors converge in the regulation of key cellular processes, including oxidative stress and hypoxia (*Nqo1*, *Keap1*, *Mafk*, *Mafg*, *Arnt*, *Arnt2*, *Vhl*, *Hif3a*), proteostasis and stress response (*Hsf2*, *Hsf2bp*, *Atg7*, *Chd6*), inflammation and transcriptional regulation (*Ep300*, *Pias4*, *Commd1*, *Cul2*, *Pitx2*), and apoptosis/cell survival (*Pin1*, *Sumo1*, *Eif2ak3*). Collectively, these findings suggest that Nrf2, HSF1, and HIF-1α act as central nodes integrating these molecular pathways, coordinating critical gene networks potentially implicated in neurodegeneration.

To assess whether these associations are maintained at the protein level, we performed an interaction analysis using STRING. The results showed a strong relationship reported in the literature (textmining) between Nrf2 (Nfe2l2), HSF1, and HIF-1α, along with a specific co-expression between Nrf2 and HIF-1α (Figure 7B). When the STRING network was expanded to 100 possible interactions within the *Mus musculus* proteome, 100 proteins with probabilities above 70% were identified. These largely overlapped with the genes previously detected in GeneMANIA and additionally highlighted other proteins linked to the same processes: oxidative stress and hypoxia (Hmox1, Egln1–3, Hif1an), proteostasis/UPR (Hsp90aa1, Hspa1b, Bag3, Eif2ak3, Xbp1), inflammation (Il1b, Il6, Ptgs2, Stat3), and apoptosis/survival (Bcl2, Casp3, Bnip3), among others (Supplementary Figure S1).

Finally, with this network of 100 proteins associated with Nrf2, HSF1, and HIF-1α, we performed a functional enrichment analysis using Gene Ontology (biological processes; Figure 7C) and Reactome (Figure 7D). Both analyses confirmed that the network is significantly associated with responses to oxidative stress, chemical stress, hypoxia, and the handling of misfolded proteins, among others.

## 3. Discussion

In this study, the neuroprotective effect of the aqueous extract of *B. procumbens* and the NAPEL formulation was evaluated in a murine model of MPTP-induced parkinsonism. MPTP administration caused motor impairment and neuronal damage in the substantia nigra, reflected by a reduction in the number of events, total distance traveled, and latency to fall in the rotarod test, as well as by a significant decrease in both the number and diameter of cells, consistent with the findings reported by Meredith et al. [36] and Wada et al. [37]. Treatment with L-DOPA significantly improved these parameters, as previously described [38]. Among the treatments evaluated, *B. procumbens* exhibited the greatest efficacy in reversing behavioral deficits and preserving cellular structure, whereas NAPEL also exerted positive effects, albeit to a lesser extent. These findings suggest that both treatments confer functional and structural protection against MPTP-induced damage, highlighting the aqueous extract of *B. procumbens* as the most effective.

Our results are consistent with those reported by Abushouk et al. [39], who documented that several plant extracts improve motor function and reduce neuronal damage in murine models of MPTP-induced parkinsonism through antioxidant and neuroprotective mechanisms. For instance, Xu et al. [40] demonstrated that the aqueous extract of ginseng improved motor behavior and promoted neuronal protection in this same model. Similarly, Chang et al. [41] observed that the extract of *Sophora tomentosa* significantly enhanced motor performance and exerted structural protective effects in affected brain regions. In line with these findings, our study shows that the aqueous extract of *B. procumbens* improved both functional and morphological outcomes in treated mice, further supporting its therapeutic potential against MPTP-induced damage.

Furthermore, we evaluated the NAPEL formulation, composed of the three major metabolites identified in the aqueous extract of *B. procumbens*: Naringenin [42], Apigenin [43], and Paeoniflorin [44], complemented with (−)-Epicatechin [45] and Lupeol [46]. These compounds have demonstrated antioxidant, anti-inflammatory, and anti-apoptotic properties; therefore, their combination aimed to explore a possible synergistic effect against MPTP-induced damage.

Our results showed that NAPEL was effective in counteracting the damage, as evidenced by significant improvements in motor behavior and histological indicators of cellular protection. These findings confirm its neuroprotective capacity in PD; however, its efficacy was slightly lower than that of the aqueous extract of *B. procumbens*, suggesting that additional, non-major metabolites in the extract may act synergistically to enhance its overall effect.

At the molecular level, we found that the neuroprotective effect of the *B. procumbens* extract against MPTP-induced damage is associated with a robust activation of the endogenous antioxidant system, primarily mediated by the Keap1/Nrf2/ARE pathway. This effect was evidenced by a significant increase in total Nrf2 expression and its active form (Nrf2-pS40), indicating its functional activation. In addition, we observed a positive regulation of key antioxidant enzymes containing ARE elements in their promoters and direct targets of Nrf2 [47], such as HO-1, CAT, and GSR, reinforcing the role of the extract in enhancing cellular defense. This pathway has previously been associated with neuroprotective effects in PD models. For instance, Williamson et al. [48] demonstrated that inhibition of Keap1 by siRNA activates the Nrf2/ARE pathway, reduces oxidative stress, and provides protection against MPTP-induced dopaminergic damage in the striatum of mice, highlighting the importance of Nrf2 activation in modulating this pathway.

Another relevant study further supports the key role of the Keap1/Nrf2/ARE pathway in neuroprotection against MPTP-induced damage: it has been demonstrated that certain small synthetic activators of this pathway reduce dopaminergic neurodegeneration in murine models of PD by increasing the expression of antioxidant enzymes and decreasing oxidative stress [49]. Our results are consistent with this mechanism, as the *B. procumbens* extract activated the Keap1/Nrf2/ARE antioxidant pathway, which was reflected in behavioral improvements and histological protection in the substantia nigra. These findings underscore that Nrf2 activation may represent a viable therapeutic strategy for PD [50].

Several studies highlight the crucial role of Nrf2-modulated antioxidant enzymes in neuroprotection against MPTP. Innamorato et al. [51] demonstrated that the absence of Nrf2 or its target gene HO-1 increases susceptibility to dopaminergic damage. Furthermore, Abdeen et al. [52] reported that alterations in SOD1 activity contribute to dopaminergic neuronal degeneration in a murine model of Parkinson’s disease. Likewise, Hussain et al. [53] showed that reduced catalase activity significantly enhances MPP^+^-induced toxicity. Similarly, Kaidery et al. [49] observed that Nrf2 activation promotes GSR expression, reducing oxidative stress and neurodegeneration. These findings support our results, which not only demonstrate the activation of Nrf2 by *B. procumbens* but also show the significant upregulation of antioxidant enzymes such as HO-1, SOD1, CAT, and GSR in both the striatum and the substantia nigra.

Complementarily, it has been reported that the transcription factor HSF1 plays a critical role in the MPTP model, exerting neuroprotective effects through the induction of heat shock proteins (HSPs) and modulation of pathways associated with cell survival and oxidative stress responses. Its activation contributes to preserving neuronal integrity and mitigating dopaminergic damage induced by the neurotoxin [54]. Furthermore, Ekimova et al. [55] demonstrated that the pharmacological activation of HSF1 with the echinochrome-derived compound U-133 increased HSP70 levels, prevented α-synuclein aggregation, microglial activation, and neuronal loss in the substantia nigra, while also reversing motor impairments characteristic of the Parkinson’s model.

In our investigation, we observed a significant increase in this protein in its monomeric, dimeric, and trimeric conformations in the striatum. In the substantia nigra, the monomeric and dimeric forms were particularly prominent, with both brain regions showing higher levels compared to all treatment groups. This profile suggests a functionally relevant activation, possibly mediated by mechanisms complementary to the Keap1/Nrf2/ARE antioxidant pathway, such as the regulation of heat shock proteins (HSPs) and modulation of the cellular stress response, contributing to the observed neuroprotective effect. Notably, when assessing lipid peroxidation through 4-HNE levels, we found a significant decrease in both brain regions of MPTP-injured mice treated with our plant extract, reinforcing the synergistic participation of the Keap1/Nrf2/ARE pathway, antioxidant enzymes, and HSF1 in neuroprotection.

Additionally, we observed that *B. procumbens* extract increased HIF-1α levels specifically in the striatum and substantia nigra of MPTP-injured mice, without affecting the levels of AKT, AKT-pS473), or β-catenin. This differential expression suggests a selective activation of stress response pathways rather than classical cell survival routes, highlighting the joint participation of Nrf2, HSF1, and HIF-1α in neuroprotection. Supporting this idea, Fujimaki et al. [56] demonstrated that activation of HIF-1α by the prolyl hydroxylase inhibitor FG-4592 in a PD model overexpressing α-synuclein promoted a comprehensive cytoprotective response. Specifically, this activation induced HO-1 expression, leading to a significant reduction in oxidative stress and protection against α-synuclein-induced neurotoxicity. In addition, a positive regulation of mitochondrial biogenesis and cellular respiration was observed, suggesting that HIF-1α functions not only as a regulator of the antioxidant response but also as a key modulator of neuronal energy metabolism, further reinforcing its relevance as a complementary mechanism in neuroprotection.

Three of the proteins evaluated in our study—Nrf2, HSF1, and HIF-1α—stand out as transcription factors that regulate a broad repertoire of genes associated with cytoprotection. These proteins may exert a synergistic and coordinated effect against oxidative stress. It has been documented that Nrf2 and HSF1 modulate cytoprotective responses through the induction of antioxidant enzymes, chaperones, and cellular repair systems, in addition, they display cross-regulation and organizational overlap by sharing common target genes such as HO-1, p62, and HSP70, which even allows them to compensate for each other [17,57]. Together with HIF-1α, these factors form a coordinated system that regulates antioxidant defense, protein quality control, and metabolic adaptation to hypoxia. Moreover, they are involved in the modulation of inflammation and apoptosis, either by suppressing inflammatory responses (Nrf2), promoting immunosurveillance (HSF1), or regulating cytokines and apoptotic pathways (HIF-1α), as reported by Cyran et al. [58].

In fact, our in silico analysis using GeneMANIA and STRING is consistent with this evidence, as it suggests that Nrf2, HSF1, and HIF-1α do not act in isolation, but rather as central nodes integrating multiple cellular processes, including oxidative stress, hypoxia, proteostasis, inflammation, and apoptosis. This convergence within interaction networks supports the idea that the observed neuroprotection does not rely exclusively on the activation of a single pathway (such as the Keap1/Nrf2/ARE axis), but on the cooperation of several stress-response axes. In this regard, the possibility that these transcription factors constitute an integrated regulatory axis (Nrf2/HSF1/HIF-1α) is particularly relevant, as it may explain the molecular synergy underlying the functional and structural protection observed in our model. Furthermore, previous studies have demonstrated that these proteins share target genes and exhibit cross-regulatory mechanisms [17,57], reinforcing the hypothesis that their coordinated action represents a critical node in neuronal resilience against neurodegeneration.

Regarding NAPEL, although it showed favorable results, it did not significantly modulate the Keap1/Nrf2/ARE pathway or most antioxidant enzymes in response to MPTP-induced damage, with the exception of an increase in GSR expression and HSF1 monomer levels in the substantia nigra. While no reduction in lipid peroxidation was detected in the striatum, a considerable decrease was observed in the substantia nigra. Despite this partial modulation, NAPEL significantly improved motor performance and preserved cellular integrity at the histological level, including cell number, size, and diameter. These findings suggest that its neuroprotective effect may be mediated by alternative and complementary mechanisms to the classical antioxidant response, such as the regulation of inflammation, mitochondrial stability, or anti-apoptotic pathways.

These findings suggest that its neuroprotective effect may be mediated by alternative and complementary mechanisms to the classical antioxidant response, such as the regulation of inflammation, mitochondrial stability, or anti-apoptotic pathways. This hypothesis is reinforced by the observation of a response profile similar to that induced by L-DOPA, which, despite not significantly modulating the levels of Nrf2, HSF1, HIF-1α, antioxidant enzymes, or reducing 4-HNE levels, exhibited the best behavioral performance in the rotarod, number of events, and open-field displacement tests. Moreover, a remarkable preservation of cellular integrity was observed in the histological analyses, suggesting that its neuroprotective effect is mediated by molecular pathways distinct from the classical antioxidant response.

In fact, Bhattacharjee et al. [59] reported that, in an MPTP-induced murine model of PD, treatment with L-DOPA increased homocysteine levels in the substantia nigra without exacerbating dopaminergic neuronal loss, suggesting neuroprotective routes independent of classical redox control. Furthermore, Chen et al. [38] demonstrated that L-DOPA treatment improved behavioral deficits in the same murine model by increasing tyrosine hydroxylase levels and reducing NLRP3 inflammasome activation in both the striatum and substantia nigra. This modulation included the downregulation of ASC, active caspase-1, IL-1β, and IL-18, suggesting that part of the neuroprotective effect of L-DOPA may be attributed to its ability to attenuate inflammasome-mediated neuroinflammation and apoptosis.

Given that the aqueous extract of *B. procumbens* exhibited the strongest neuroprotective effect, it is reasonable to consider that, in addition to the major metabolites, minor constituents also contribute to the observed mechanisms. This is evidenced by the fact that the NAPEL formulation, composed of the major metabolites of the extract, displayed a weaker effect than the whole extract, suggesting a synergistic contribution of both major and minor compounds in *B. procumbens*.

In our research group, HPLC analysis identified naringenin (29.2%), equol 7-O-glucuronide (19.3%), and paeoniflorin (8.2%) as the main compounds of the extract, which together accounted for 56.7% of the total profile [30]. However, other metabolites such as Ferulic acid (5.0%), Acanthoside B (4.2%), 4-Hydroxybenzoic acid (3.6%), and Genistein (1.0%) have also been reported as neuroprotective agents in the MPTP-induced parkinsonism model [60,61,62,63]. This suggests that the synergistic action between major and minor metabolites contributes to the superior performance of the extract compared with the NAPEL formulation.

Two of the three major metabolites of the extract have been widely documented as neuroprotective in the murine Parkinson’s model induced by MPTP. Naringenin, the most abundant compound, reduces lipid peroxidation, increases the activity of antioxidant enzymes such as glutathione reductase and catalase, improves motor performance, and attenuates histological damage in the substantia nigra and striatum [42]. Paeoniflorin, in turn, preserves dopaminergic integrity through the regulation of dopamine metabolism, inhibition of the Bcl-2/Bax/caspase apoptotic pathway, and possible activation of PI3K/AKT, in addition to significantly improving motor function [64].

Similarly, other metabolites of the extract have also shown neuroprotective properties in this model. Ferulic acid protects against oxidative stress, modulates neuroinflammation, and preserves mitochondrial function [60]. Acanthoside improves motor performance, preserves striatal dopamine levels, increases TH immunoreactivity in the substantia nigra, and positively regulates α-synuclein, suggesting a protective effect against dopaminergic damage [61]. 4-Hydroxybenzoic acid attenuates oxidative stress and excitotoxicity while modulating the microglial inflammatory response [62]. Finally, Genistein restores dopamine levels and its metabolites, preserves TH-positive neurons in the substantia nigra, and increases the expression of DAT and Bcl-2, demonstrating an anti-apoptotic mechanism [63].

Although NAPEL and the *B. procumbens* extract share key metabolites such as naringenin and paeoniflorin, their effects likely differed due to variations in concentration, bioavailability, or the presence of other minor compounds in the extract that act synergistically, generating a more robust neuroprotective response.

**Figure 8:**
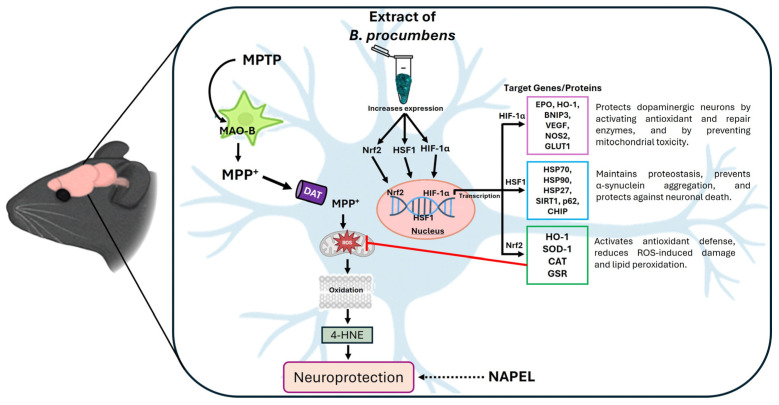
Schematic representation of the proposed neuroprotective mechanism for *B. procumbens*. The extract primarily acts through the activation of the transcription factors Nrf2, HSF1, and HIF-1α, which regulate target genes involved in antioxidant defense, proteostasis, and cellular adaptation to stress [24,65,66]. This coordinated regulation reduces MPTP-induced oxidative damage (as evidenced by 4-HNE levels), thereby promoting neuroprotection. Although the NAPEL formulation did not share the same mechanism observed with *B. procumbens*, it still produced cytoprotective effects. Therefore, it is represented with a dashed arrow indicating its potential neuroprotective action through mechanisms that remain to be elucidated.

In summary, Figure 8 presents a proposed molecular model illustrating the potential mechanisms through which the *B. procumbens* extract exerts its neuroprotective effect.

### Limitations and Perspectives

The MPTP model reflects acute neurotoxicity rather than the chronic progression of PD, and only selected cytoprotective pathways were evaluated, leaving other relevant mechanisms unexplored. Additionally, the interactomic analysis is predictive and requires further experimental validation.

Currently, complementary studies are being conducted to investigate the mechanisms of action of *B. procumbens* and NAPEL, including markers of inflammation, apoptosis, α-synuclein aggregation, dopamine transporters, and tyrosine hydroxylase. These analyses, together with future studies in chronic PD models and assessments of pharmacokinetics and bioavailability, will help validate the in silico-predicted nodes and more precisely define the neuroprotective mechanisms and translational potential of these treatments.

## 4. Materials and Methods

### 4.1. Procurement of Bioactive Compounds

The aqueous extract of *B. procumbens* was obtained following the protocol described by Martínez-Cuazitl et al. [30] using dried and ground plant material cultivated in a greenhouse under controlled conditions. The greenhouse was equipped with an anti-aphid mesh, a white polyethylene roof (30% shading), and a substrate composed of perlite and organic matter. Irrigation was performed automatically every 3 h during spring–summer and every 12 h in winter, maintaining a relative humidity of 67% and average temperatures of 38 °C in spring–summer and 29 °C in winter. Extraction was carried out by reflux with an ethanol–water solution (50:50) at 50 °C for 4 h, and the process was repeated three times. Subsequently, the extract was concentrated under vacuum and lyophilized using a Telstar^®^ system (Terrassa, Barcelona, Spain).

The components of the NAPEL formulation, along with the doses employed and the solvents used, are detailed in Table 1. The name NAPEL corresponds to the initials of the active metabolites comprising the formulation: **N**aringenin, **A**pigenin, **P**aeoniflorin, (−)-**E**picatechin, and **L**upeol. The selection of these compounds was based on the three most abundant metabolites identified in the aqueous extract of *B. procumbens* (N, A, and P), to which E and L were added due to evidence supporting their neuroprotective potential reported in various preclinical models of PD [42,43,44,45,46].

### 4.2. Experimental Animals

Male C57BL/6 mice, 8 weeks of age and body weight of 25 ± 2 g, were used in this study. The animals were housed in an animal facility under controlled environmental conditions: temperature of 22 °C, relative humidity of 55 ± 5%, and a 12 h light/dark cycle. They had ad libitum access to water and a balanced diet and were maintained in a room exclusively designated for experimental animals. The experimental protocol was approved by the Bioethics Committee of ENMH-IPN (registration number CBE/005/2021) and was conducted in accordance with the Mexican Official Standard NOM-062-ZOO-1999 and the Guide for the Care and Use of Laboratory Animals of the National Research Council [67].

### 4.3. Experimental Model of MPTP-Induced Parkinson’s Disease

The experimental model was performed according to the protocol described by Wu et al. [68] and consisted of an acute procedure for inducing neurological damage through the administration of MPTP (Sigma-Aldrich, St. Louis, MO, USA).

Animals were randomly assigned to six experimental groups (*n* = 6 per group):

1. Healthy control: treated only with saline solution.
2. *B. procumbens* (Bp) control: treated with the aqueous extract of *B. procumbens* (two doses of 150 mg/kg).
3. MPTP: neurological damage induced by MPTP without additional treatment.
4. MPTP+L-DOPA: neurological damage induced by MPTP and treated with levodopa.
5. MPTP+Bp: neurological damage induced by MPTP and treated with extract of *B. procumbens*.
6. MPTP+NAPEL: neurological damage induced by MPTP and treated with formulation NAPEL.

The treatment doses were as follows: MPTP (18 mg/kg) was used to induce neurological damage, while L-DOPA (50 mg/kg) and the aqueous extract of *B. procumbens* (150 mg/kg) were administered as treatments, all dissolved in sterile saline solution. The selected dose of the *B. procumbens* extract was based on our previous studies in different experimental models, where this concentration consistently showed the best therapeutic performance and was therefore chosen for the present study.

The metabolites of the NAPEL formulation were prepared and dissolved as described in Table 1. The healthy control group received only saline. In all cases, administrations were performed intraperitoneally (i.p.) at a total volume of 200 μL per dose.

**Figure 9:**
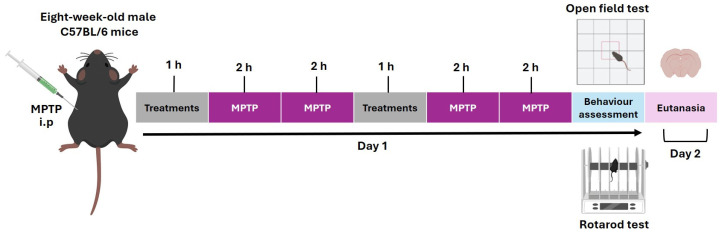
Timeline of the experimental protocol with MPTP and treatment. The sequence of toxin injections, treatment administration, behavioral evaluation, and sacrifice are shown. i.p., intraperitoneal route of administration.

Our experimental design lasted 2 days [68]. On the first day, an initial dose of the corresponding treatment (L-DOPA, *B. procumbens*, or NAPEL) was administered, and after an interval of 1 h, the first two MPTP injections were given at 2 h intervals. Subsequently, a second dose of the treatment was administered, and 1 h later, two additional MPTP injections were applied, also at 2 h intervals. Finally, 1 h after the last MPTP injection, behavioral evaluation was performed. On the second day (24 h later), the animals were euthanized by cervical dislocation for the collection of biological samples (Figure 9).

### 4.4. Motor Function Tests: Open Field and Rotarod

At the end of the treatment period, motor function was evaluated through a series of behavioral assessments widely used in Parkinson’s disease models [69]. Locomotor and exploratory activities were assessed in the open field test [70,71]. Briefly, each mouse was individually placed in an acrylic arena (50 × 50 × 50 cm) equipped with a video-tracking and behavioral analysis system (OmniAlva^®^, version 2.0.0). Each session lasted 5 min, and the arena was disinfected with 80% ethanol between trials to avoid olfactory cues. The parameters recorded included total distance traveled (cm), movement trajectory, and number of rearing episodes (standing on the hind limbs), which reflect spontaneous locomotion and exploratory behavior.

Motor coordination, balance, and fatigue resistance were assessed by the rotarod test [72,73]. Mice were placed individually on a rotating rod (3 cm in diameter) at a constant speed of 5 rpm. Three consecutive trials per animal were performed, separated by a 2 min rest interval. The latency to fall was recorded as a measure of motor performance. To minimize stress and ensure proper adaptation, a training session was conducted one day before the evaluation.

### 4.5. Luxol Fast Blue Staining

After euthanasia, brains were extracted and fixed in 4% paraformaldehyde at 4 °C for 24 h. The tissues were then processed for 16 h in a MICROM/STP120-1 tissue processor (Thermo Scientific, Walldorf, Germany) and embedded in Paraplast paraffin blocks (McCormick; Medex Supply, Brooklyn, NY, USA).

Coronal sections of 6 µm thickness were obtained, mounted on glass slides, and stained with Luxol Fast Blue (LFB, Sigma-Aldrich, St. Louis, MO, USA) to evaluate myelination. Sections were deparaffinized in xylene and rehydrated through a descending alcohol series (100%, 95%, 70%) and finally distilled water.

The sections were incubated in 0.1% LFB dissolved in 95% ethanol for 24 h at 56–60 °C. They were subsequently differentiated in 0.05% lithium carbonate and 70% alcohol until optimal contrast was achieved between white matter (intensely blue) and gray matter.

Finally, sections were dehydrated, cleared in xylene, and mounted with permanent mounting medium. Images were captured using optical microscopy with an Olympus microscope (Tokyo, Japan) equipped with a DP21 photographic system.

### 4.6. Protein Extraction

On the day of euthanasia, brain dissections were performed on mice from each experimental group, specifically isolating the striatum and substantia nigra. Tissues were immediately placed in RIPA buffer (50 mM Tris-HCl, pH 7.5; 150 mM NaCl; 1% NP-40; 0.1% SDS; 1 mM EDTA) supplemented with a Complete™ protease inhibitor cocktail (Merck, Darmstadt, Hesse, Germany). Tissue homogenization was carried out for 3 min at 5000 rpm using a tissue homogenizer (Ultra-Turrax T18 digital, IKA^®^, San Diego, CA, USA).

Subsequently, the homogenates were centrifuged at 10,000 rpm for 15 min at 4 °C, and the supernatant corresponding to the soluble protein fraction was collected. Protein quantification was performed using the Bradford method [74].

### 4.7. Western Blotting

A total of 20 µg of protein per sample were loaded onto a 12% polyacrylamide gel (SDS-PAGE) for electrophoretic separation, followed by transfer of proteins onto polyvinylidene difluoride (PVDF) membranes (Merck, Darmstadt, HE, Germany). Membranes were blocked with 2% bovine serum albumin (BSA) dissolved in PBS (pH 7.4) for 1 h at room temperature to prevent nonspecific binding.

Subsequently, membranes were incubated overnight at 4 °C under constant agitation with all primary antibodies, each diluted 1:20,000 in PBS containing 0.05% BSA and 0.05% Tween. After six washes of 20 min each with PBS, membranes were incubated with horseradish peroxidase (HRP)-conjugated secondary antibodies (1:45,000 dilution) for 30 min at room temperature. Following this incubation, six additional washes of 20 min each were performed. Protein detection was carried out using the Immobilon Western chemiluminescent kit (Millipore-Merck, Billerica, MA, USA), according to the manufacturer’s instructions.

The primary antibodies used were: anti-Nrf2 (GeneTex, Irvine, CA, USA), anti-p-Nrf2-pS40 (anti-Nrf2 phosphorylated at serine 40, GeneTex), anti-heme oxygenase-1 (HO-1) (Santa Cruz Biotechnology, Dallas, TX, USA; sc-374436, sc-39991), anti-SOD1 (Santa Cruz Biotechnology), anti-catalase (Santa Cruz Biotechnology), anti-glutathione reductase (Santa Cruz Biotechnology), anti-HSF1 (Santa Cruz Biotechnology), anti-4-HNE (GeneTex), HIF-1α (Santa Cruz Biotechnology), anti-Akt (GeneTex), anti-AKT-pS473 (anti-AKT phosphorylated at serine 473, GeneTex), β-catenin (GeneTex), and Anti-GAPDH (GeneTex) was used as an endogenous control.

The secondary antibodies used were mouse anti-IgG (Jackson ImmunoResearch, West Grove, PA, USA) or rabbit anti-IgG (Jackson ImmunoResearch), both conjugated to HRP and selected according to the species of the primary antibody.

### 4.8. Interactome and Functional Enrichment Analysis of Nrf2, HSF1, and HIF-1α

The methodological strategy for interactome and functional enrichment analysis was conducted following criteria similar to those reported by Pérez et al. [33], in order to ensure robustness and reproducibility of the results. Gene interaction networks were constructed using the GeneMANIA server, version 2025 (https://genemania.org/, accessed on 24 August 2025) [75]. The analysis focused on the genes encoding the transcription factors Nrf2 (Nfe2l2), HSF1, and HIF-1α, integrating gene-level interactions in *Mus musculus*. A network of the 20 most associated genes was obtained, considering evidence from co-expression, shared metabolic pathways, protein domains, and literature data, which enabled the identification of the initial functional associations.

Subsequently, to corroborate potential connections between these transcription factors at the protein level, the STRING platform, version 12 (https://string-db.org/, accessed on 25 August 2025), was employed. In this case, both known interactions (from curated databases and experimental data) and predicted interactions (gene neighborhood, gene fusions, gene co-occurrence, text mining, co-expression, and protein homology) were considered [76,77]. This analysis yielded a primary network restricted to Nrf2, HSF1, and HIF-1α.

The network was then expanded to the *M. musculus* proteome, limiting the associations to the 100 proteins with the highest interaction potential. Finally, functional enrichment analysis was performed using STRING-integrated tools: Gene Ontology (biological processes) and Reactome pathway enrichment. Both analyses allowed the identification of the main molecular pathways associated with the transcription factors Nrf2, Hsf1, and HIF-1α.

In both GeneMANIA and STRING, the search input consisted of the terms “Nrf2, HSF1, and HIF1a,” specifying *M. musculus* as the species. All analyses were conducted applying a probability threshold of ≥70%.

### 4.9. Statistical Analysis and Graphical Representation

Data were analyzed using two-way ANOVA followed by Tukey’s post hoc test for multiple comparisons. Significance levels were reported following the American Psychological Association (APA) format. For all symbols (¡, *, &), statistical significance was defined as follows: one symbol = *p* ≤ 0.033; two symbols = *p* ≤ 0.002; three symbols = *p* ≤ 0.001. Results are expressed as mean ± standard deviation (SD), based on six mice per experimental group. Graphical representations were generated using GraphPad Prism version 8.0.1 (GraphPad Software, San Diego, CA, USA).

## 5. Conclusions

The constituent metabolites of the aqueous extract of *B. procumbens* exert a significant neuroprotective effect against MPTP-induced damage, mediated by the activation of the transcription factors Nrf2, HSF1, and HIF-1α, as well as by the induction of antioxidant enzymes and the reduction in lipid peroxidation. In contrast, although NAPEL demonstrated neuroprotective effects at the behavioral and histological levels, it did not reproduce the same molecular profile as the extract. This indicates that the efficacy of the extract is not limited to its major metabolites but likely results from the synergy with minor compounds, which may explain its superiority over the defined formulation. Therefore, the aqueous extract of *B. procumbens* and the NAPEL formulation emerge as promising candidates for the development of novel therapeutic strategies against Parkinson’s disease.

## Figures and Tables

**Table 1:** Components of the NAPEL formulation.

Metabolite	Administered Dose (mg/kg)	Solvent	References
Naringenin	10	DMSO 10%	Sugumar et al. [42]
Apigenin	10	DMSO 20%	Yarim et al. [43]
Paeoniflorin	10	Water	Wang et al. [44]
(−)-Epicatechin	3	DMSO 1%	Zhou et al. [45]
Lupeol	6	DMSO 20%	Ahmad et al. [46]

Dimethyl sulfoxide; DMSO.

## Data Availability

The original contributions presented in this study are included in the article/Supplementary Material. Further inquiries can be directed to the corresponding author.

## Supplementary Materials

The following supporting information can be downloaded at: https://www.mdpi.com/article/10.3390/ijms262411914/s1.

## Abbreviations

The following abbreviations are used in this manuscript:

Abbreviation	Name
Nrf2	Nuclear factor erythroid 2–related factor 2
Nrf2-pS40	Phosphorylated Nrf2 at serine 40
HO-1	Heme oxygenase-1
SOD1	Superoxide dismutase 1
CAT	Catalase
GSR	Glutathione-disulfide reductase
HSF1	Heat shock factor 1
HIF-1α	Hypoxia-inducible factor 1-alpha
AKT	Protein kinase B (PKB/AKT)
AKT-pS473	Phosphorylated AKT at serine 473
β-catenin	Beta-catenin
4-HNE	4-Hydroxy-2-nonenal
DAT	Dopamine transporter
MAO-B	Monoamine oxidase B
KEAP1	Kelch-like ECH-associated protein 1
Mafk	Transcription factor MafK
Arnt	Aryl hydrocarbon receptor nuclear translocator
Vhl	Von Hippel–Lindau tumor suppressor
Hsf2bp	Heat shock factor 2 binding protein
Atg7	Autophagy related 7
Chd6	Chromodomain-helicase-DNA-binding protein 6
Ep300	E1A binding protein p300
Pias4	Protein inhibitor of activated STAT 4
Commd1	COMM domain-containing protein 1
Cul2	Cullin-2
Pitx2	Paired-like homeodomain transcription factor 2
Pin1	Peptidyl-prolyl cis-trans isomerase NIMA-interacting 1
Sumo1	Small ubiquitin-like modifier 1
Eif2ak3	Eukaryotic translation initiation factor 2-alpha kinase 3 (also known as PERK)
Hmox1	Heme oxygenase 1 (same as HO-1)
Egln1–3	Prolyl hydroxylase domain-containing proteins 1–3 (EGLN1, EGLN2, EGLN3)
Hif1an	Hypoxia-inducible factor 1-alpha inhibitor
Hspa1b	Heat shock protein family A member 1B (HSP70 family)
Bag3	BCL2-associated athanogene 3
Xbp1	X-box binding protein 1
GAPDH	Glyceraldehyde-3-phosphate dehydrogenase

## References

[1] Estadísticas|Parkinson’s Foundation. https://www.parkinson.org/espanol/entendiendo-parkinson/estadisticas.

[2] Mhyre T.R., Boyd J.T., Hamill R.W., Maguire-Zeiss K.A. (2012). Parkinson’s Disease. Subcell. Biochem..

[3] Stocchi F., Bravi D., Emmi A., Antonini A. (2024). Parkinson Disease Therapy: Current Strategies and Future Research Priorities. Nat. Rev. Neurol..

[4] Kouli A., Torsney K.M., Kuan W.-L., Stoker T.B., Greenland J.C. (2018). Parkinson’s Disease: Etiology, Neuropathology, and Pathogenesis. Parkinson’s Disease: Pathogenesis and Clinical Aspects.

[5] Beheshti I. (2025). Exploring Risk and Protective Factors in Parkinson’s Disease. Cells.

[6] Chakrabarti S., Bisaglia M. (2023). Oxidative Stress and Neuroinflammation in Parkinson’s Disease: The Role of Dopamine Oxidation Products. Antioxidants.

[7] Mustapha M., Taib C.N.M. (2021). MPTP-Induced Mouse Model of Parkinson’s Disease: A Promising Direction for Therapeutic Strategies. Bosn. J. Basic Med. Sci..

[8] Mondal S., Firdous S.M. (2025). Unrevealing the Molecular Mechanisms of MPTP-Induced Parkinson’s in Experimental Animals. Med. Chem. Res..

[9] Pardo-Moreno T., García-Morales V., Suleiman-Martos S., Rivas-Domínguez A., Mohamed-Mohamed H., Ramos-Rodríguez J.J., Melguizo-Rodríguez L., González-Acedo A. (2023). Current Treatments and New, Tentative Therapies for Parkinson’s Disease. Pharmaceutics.

[10] Velázquez-Paniagua M., Vázquez-Álvarez A.M., Valverde-Aguilar G., Vergara-Aragón P. (2016). Current Treatments in Parkinson’s Including the Proposal of an Innovative Dopamine Microimplant. Rev. Médica Hosp. Gen. México.

[11] Kaur T., Sidana P., Kaur N., Choubey V., Kaasik A. (2024). Unraveling Neuroprotection in Parkinson’s Disease: Nrf2–Keap1 Pathway’s Vital Role amidst Pathogenic Pathways. Inflammopharmacology.

[12] Huenchuguala S., Segura-Aguilar J. (2024). Natural Compounds That Activate the KEAP1/Nrf2 Signaling Pathway as Potential New Drugs in the Treatment of Idiopathic Parkinson’s Disease. Antioxidants.

[13] Liu S., Pi J., Zhang Q. (2022). Signal Amplification in the KEAP1-NRF2-ARE Antioxidant Response Pathway. Redox Biol..

[14] Bhurtel S., Bok E., Katila N., Kim J., Choi D.-Y. (2021). Activation of Nrf2 by Methylene Blue Is Associated with the Neuroprotection against MPP+ Induced Toxicity via Ameliorating Oxidative Stress and Mitochondrial Dysfunction. Biochem. Pharmacol..

[15] Chen H., Ma D., Yue F., Qi Y., Dou M., Cui L., Xing Y. (2022). The Potential Role of Hypoxia-Inducible Factor-1 in the Progression and Therapy of Central Nervous System Diseases. Curr. Neuropharmacol..

[16] Qu Z., Titus A.S.C.L.S., Xuan Z., D’Mello S.R. (2018). Neuroprotection by Heat Shock Factor-1 (HSF1) and Trimerization-Deficient Mutant Identifies Novel Alterations in Gene Expression. Sci. Rep..

[17] Dayalan Naidu S., Kostov R.V., Dinkova-Kostova A.T. (2015). Transcription Factors Hsf1 and Nrf2 Engage in Crosstalk for Cytoprotection. Trends Pharmacol. Sci..

[18] Acuña-Pilarte K., Koh M.Y. (2025). The HIF Axes in Cancer: Angiogenesis, Metabolism, and Immune-Modulation. Trends Biochem. Sci..

[19] Zhang J., Yao M., Xia S., Zeng F., Liu Q. (2025). Systematic and Comprehensive Insights into HIF-1 Stabilization under Normoxic Conditions: Implications for Cellular Adaptation and Therapeutic Strategies in Cancer. Cell. Mol. Biol. Lett..

[20] Lee J.-W., Bae S.-H., Jeong J.-W., Kim S.-H., Kim K.-W. (2004). Hypoxia-Inducible Factor (HIF-1)α: Its Protein Stability and Biological Functions. Exp. Mol. Med..

[21] Vladimirova S.A., Kokoreva N.E., Guzhova I.V., Alhasan B.A., Margulis B.A., Nikotina A.D. (2024). Unveiling the HSF1 Interaction Network: Key Regulators of Its Function in Cancer. Cancers.

[22] Pérez-Mora S., Pérez-Ishiwara D.G., Salgado-Hernández S.V., Medel-Flores M.O., Reyes-López C.A., Rodríguez M.A., Sánchez-Monroy V., Gómez-García M.d.C. (2024). Entamoeba Histolytica: In Silico and In Vitro Oligomerization of EhHSTF5 Enhances Its Binding to the HSE of the EhPgp5 Gene Promoter. Int. J. Mol. Sci..

[23] Dorantes-Palma D., Pérez-Mora S., Azuara-Liceaga E., Pérez-Rueda E., Pérez-Ishiwara D.G., Coca-González M., Medel-Flores M.O., Gómez-García C. (2024). Screening and Structural Characterization of Heat Shock Response Elements (HSEs) in Entamoeba Histolytica Promoters. Int. J. Mol. Sci..

[24] Chowdhury R., Hardy A., Schofield C.J. (2008). The Human Oxygen Sensing Machinery and Its Manipulation. Chem. Soc. Rev..

[25] Sharma S.K., Priya S. (2016). Expanding Role of Molecular Chaperones in Regulating α-Synuclein Misfolding; Implications in Parkinson’s Disease. Cell. Mol. Life Sci. CMLS.

[26] Tanaka K., Galduróz R.F.S.-, Gobbi L.T.B., Galduróz J.C.F. (2013). Ginkgo Biloba Extract in an Animal Model of Parkinson’s Disease: A Systematic Review. Curr. Neuropharmacol..

[27] Patel A., Olang C.A., Lewis G., Mandalaneni K., Anand N., Gorantla V.R. (2022). An Overview of Parkinson’s Disease: Curcumin as a Possible Alternative Treatment. Cureus.

[28] Huang J., Zhang X., Yang X., Yv Q., Ye F., Chen S., Cui Y., Gu L., Zhu M., Li W. (2024). Baicalin Exerts Neuroprotective Actions by Regulating the Nrf2-NLRP3 Axis in Toxin-Induced Models of Parkinson’s Disease. Chem. Biol. Interact..

[29] Kosmopoulou D., Lafara M.-P., Adamantidi T., Ofrydopoulou A., Grabrucker A.M., Tsoupras A. (2024). Neuroprotective Benefits of Rosmarinus Officinalis and Its Bioactives against Alzheimer’s and Parkinson’s Diseases. Appl. Sci..

[30] Martínez-Cuazitl A., Gómez-García M.d.C., Hidalgo-Alegria O., Flores O.M., Núñez-Gastélum J.A., Martínez E.S.M., Ríos-Cortés A.M., Garcia-Solis M., Pérez-Ishiwara D.G. (2022). Characterization of Polyphenolic Compounds from Bacopa Procumbens and Their Effects on Wound-Healing Process. Molecules.

[31] Martínez-Cuazitl A., Gómez-García M.D.C., Pérez-Mora S., Rojas-López M., Delgado-Macuil R.J., Ocampo-López J., Vázquez-Zapién G.J., Mata-Miranda M.M., Pérez-Ishiwara D.G. (2023). Polyphenolic Compounds Nanostructurated with Gold Nanoparticles Enhance Wound Repair. Int. J. Mol. Sci..

[32] Pérez-Mora S., Ocampo-López J., Gómez-García M.D.C., Pérez-Ishiwara D.G. (2023). BFNB Enhances Hair Growth in C57BL/6 Mice through the Induction of EGF and FGF7 Factors and the PI3K-AKT-β-Catenin Pathway. Int. J. Mol. Sci..

[33] Pérez-Mora S., Ocampo-López J., Gómez-García M.d.C., Salgado-Hernández S.V., Flores-Martinez Y.M., Pérez-Ishiwara D.G. (2025). Polyphenols from Bacopa Procumbens Nanostructured with Gold Nanoparticles Stimulate Hair Growth Through Apoptosis Modulation in C57BL/6 Mice. Pharmaceutics.

[34] Nagarajan S., Chellappan D.R., Chinnaswamy P., Thulasingam S. (2015). Ferulic Acid Pretreatment Mitigates MPTP-Induced Motor Impairment and Histopathological Alterations in C57BL/6 Mice. Pharm. Biol..

[35] Kawagoe S., Matsusaki M., Mabuchi T., Ogasawara Y., Watanabe K., Ishimori K., Saio T. (2025). Mechanistic Insights into Oxidative Response of Heat Shock Factor 1 Condensates. JACS Au.

[36] Meredith G.E., Rademacher D.J. (2011). MPTP Mouse Models of Parkinson’s Disease: An Update. J. Park. Dis..

[37] Wada M., Ang M.J., Weerasinghe-Mudiyanselage P.D.E., Kim S.-H., Kim J.-C., Shin T., Moon C. (2021). Behavioral Characterization in MPTP/p Mouse Model of Parkinson’s Disease. J. Integr. Neurosci..

[38] Chen X., Wang Z., Yang W., Fu Y. (2024). Levodopa Improves Behavioral Deficits of Mice with Parkinson’s Disease Symptoms via Curbing NLRP3 Inflammasome Activation and Enhancing Tyrosine Hydroxylase Levels in the Striatum and Substantia Nigra. J. Integr. Neurosci..

[39] Abushouk A.I., Negida A., Ahmed H., Abdel-Daim M.M. (2017). Neuroprotective Mechanisms of Plant Extracts against MPTP Induced Neurotoxicity: Future Applications in Parkinson’s Disease. Biomed. Pharmacother..

[40] Xu N., Xing S., Li J., Pang B., Liu M., Fan M., Zhao Y. (2024). Water Extract of Ginseng Alleviates Parkinsonism in MPTP-Induced Parkinson’s Disease Mice. PLoS ONE.

[41] Chang H.-C., Liu K.-F., Teng C.-J., Lai S.-C., Yang S.-E., Ching H., Wu C.-R. (2019). Sophora Tomentosa Extract Prevents MPTP-Induced Parkinsonism in C57BL/6 Mice Via the Inhibition of GSK-3β Phosphorylation and Oxidative Stress. Nutrients.

[42] Sugumar M., Sevanan M., Sekar S. (2019). Neuroprotective Effect of Naringenin against MPTP-Induced Oxidative Stress. Int. J. Neurosci..

[43] Yarim G.F., Kazak F., Yarim M., Sozmen M., Genc B., Ertekin A., Gokceoglu A. (2025). Apigenin Alleviates Neuroinflammation in a Mouse Model of Parkinson’s Disease. Int. J. Neurosci..

[44] Wang L., An H., Yu F., Yang J., Ding H., Bao Y., Xie H., Huang D. (2022). The Neuroprotective Effects of Paeoniflorin against MPP+-Induced Damage to Dopaminergic Neurons via the Akt/Nrf2/GPX4 Pathway. J. Chem. Neuroanat..

[45] Zhou T., Zhu M., Liang Z. (2018). (-)-Epigallocatechin-3-Gallate Modulates Peripheral Immunity in the MPTP-Induced Mouse Model of Parkinson’s Disease. Mol. Med. Rep..

[46] Ahmad R., Khan A., Lee H.J., Ur Rehman I., Khan I., Alam S.I., Kim M.O. (2020). Lupeol, a Plant-Derived Triterpenoid, Protects Mice Brains against Aβ-Induced Oxidative Stress and Neurodegeneration. Biomedicines.

[47] Ngo V., Duennwald M.L. (2022). Nrf2 and Oxidative Stress: A General Overview of Mechanisms and Implications in Human Disease. Antioxidants.

[48] Williamson T.P., Johnson D.A., Johnson J.A. (2012). Activation of the Nrf2-ARE Pathway by siRNA Knockdown of Keap1 Reduces Oxidative Stress and Provides Partial Protection from MPTP-Mediated Neurotoxicity. Neurotoxicology.

[49] Kaidery N.A., Banerjee R., Yang L., Smirnova N.A., Hushpulian D.M., Liby K.T., Williams C.R., Yamamoto M., Kensler T.W., Ratan R.R. (2013). Targeting Nrf2-Mediated Gene Transcription by Extremely Potent Synthetic Triterpenoids Attenuate Dopaminergic Neurotoxicity in the MPTP Mouse Model of Parkinson’s Disease. Antioxid. Redox Signal..

[50] Yang X.-X., Yang R., Zhang F. (2022). Role of Nrf2 in Parkinson’s Disease: Toward New Perspectives. Front. Pharmacol..

[51] Innamorato N.G., Jazwa A., Rojo A.I., García C., Fernández-Ruiz J., Grochot-Przeczek A., Stachurska A., Jozkowicz A., Dulak J., Cuadrado A. (2010). Different Susceptibility to the Parkinson’s Toxin MPTP in Mice Lacking the Redox Master Regulator Nrf2 or Its Target Gene Heme Oxygenase-1. PLoS ONE.

[52] Abdeen A.H., Trist B.G., Nikseresht S., Harwood R., Roudeau S., Rowlands B.D., Kreilaus F., Cottam V., Mor D., Richardson M. (2025). Parkinson-like Wild-Type Superoxide Dismutase 1 Pathology Induces Nigral Dopamine Neuron Degeneration in a Novel Murine Model. Acta Neuropathol..

[53] Hussain S., Hass B.S., Slikker W., Ali S.F. (1999). Reduced Levels of Catalase Activity Potentiate MPP+-Induced Toxicity: Comparison between MN9D Cells and CHO Cells. Toxicol. Lett..

[54] Liao Y., Gu Y., Wang J., Tian Y., Ni X., Zhou L., Ye Y., Xia G. (2023). HSF1 Inhibits Microglia Activation to Attenuate Neuroinflammation via Regulating miR-214-3p and NFATc2 in Parkinson’s Disease. Folia Neuropathol..

[55] Ekimova I.V., Plaksina D.V., Pastukhov Y.F., Lapshina K.V., Lazarev V.F., Mikhaylova E.R., Polonik S.G., Pani B., Margulis B.A., Guzhova I.V. (2018). New HSF1 Inducer as a Therapeutic Agent in a Rodent Model of Parkinson’s Disease. Exp. Neurol..

[56] Fujimaki A., Ohuchi K., Takizawa S., Murakami T., Kurita H., Hozumi I., Wen X., Kitamura Y., Wu Z., Maekawa Y. (2023). The Neuroprotective Effects of FG-4592, a Hypoxia-Inducible Factor-Prolyl Hydroxylase Inhibitor, against Oxidative Stress Induced by Alpha-Synuclein in N2a Cells. Sci. Rep..

[57] Inouye S., Hatori Y., Kubo T., Saito S., Kitamura H., Akagi R. (2018). NRF2 and HSF1 Coordinately Regulate Heme Oxygenase-1 Expression. Biochem. Biophys. Res. Commun..

[58] Cyran A.M., Zhitkovich A. (2022). HIF1, HSF1, and NRF2: Oxidant-Responsive Trio Raising Cellular Defenses and Engaging Immune System. Chem. Res. Toxicol..

[59] Bhattacharjee N., Mazumder M.K., Paul R., Choudhury A., Choudhury S., Borah A. (2016). L-DOPA Treatment in MPTP-Mouse Model of Parkinson’s Disease Potentiates Homocysteine Accumulation in Substantia Nigra. Neurosci. Lett..

[60] Hassani S., Esmaeili A. (2024). The Neuroprotective Effects of Ferulic Acid in Toxin-Induced Models of Parkinson’s Disease: A Review. Ageing Res. Rev..

[61] Geng X., Tian X., Tu P., Pu X. (2007). Neuroprotective Effects of Echinacoside in the Mouse MPTP Model of Parkinson’s Disease. Eur. J. Pharmacol..

[62] Winter A.N., Brenner M.C., Punessen N., Snodgrass M., Byars C., Arora Y., Linseman D.A. (2017). Comparison of the Neuroprotective and Anti-Inflammatory Effects of the Anthocyanin Metabolites, Protocatechuic Acid and 4-Hydroxybenzoic Acid. Oxid. Med. Cell. Longev..

[63] Liu L.-X., Chen W.-F., Xie J.-X., Wong M.-S. (2008). Neuroprotective Effects of Genistein on Dopaminergic Neurons in the Mice Model of Parkinson’s Disease. Neurosci. Res..

[64] Zheng M., Liu C., Fan Y., Yan P., Shi D., Zhang Y. (2017). Neuroprotection by Paeoniflorin in the MPTP Mouse Model of Parkinson’s Disease. Neuropharmacology.

[65] Kovács D., Sigmond T., Hotzi B., Bohár B., Fazekas D., Deák V., Vellai T., Barna J. (2019). HSF1Base: A Comprehensive Database of HSF1 (Heat Shock Factor 1) Target Genes. Int. J. Mol. Sci..

[66] He F., Ru X., Wen T. (2020). NRF2, a Transcription Factor for Stress Response and Beyond. Int. J. Mol. Sci..

[67] Muñoz L.I.O. (1999). NORMA Oficial Mexicana NOM-062-ZOO-1999. Especificaciones Tecnicas Para la Produccion, Cuidado y Uso de los Animales de Laboratorio.

[68] Wu D.C., Jackson-Lewis V., Vila M., Tieu K., Teismann P., Vadseth C., Choi D.-K., Ischiropoulos H., Przedborski S. (2002). Blockade of Microglial Activation Is Neuroprotective in the 1-Methyl-4-Phenyl-1,2,3,6-Tetrahydropyridine Mouse Model of Parkinson Disease. J. Neurosci. Off. J. Soc. Neurosci..

[69] Lane E., Dunnett S. (2008). Animal Models of Parkinson’s Disease and L-Dopa Induced Dyskinesia: How Close Are We to the Clinic?. Psychopharmacology.

[70] Farfán-García E.D., Abad-García A., Alatorre A., Pérez-Capistran T., Querejeta E., Soriano-Ursúa M.A. (2020). Olive Oil Limited Motor Disruption and Neuronal Damage in Parkinsonism Induced by MPTP Administration. Toxicol. Res. Appl..

[71] Gould T.D., Dao D.T., Kovacsics C.E. (2009). The Open Field Test. Mood and Anxiety Related Phenotypes in Mice: Characterization Using Behavioral Tests.

[72] Shiotsuki H., Yoshimi K., Shimo Y., Funayama M., Takamatsu Y., Ikeda K., Takahashi R., Kitazawa S., Hattori N. (2010). A Rotarod Test for Evaluation of Motor Skill Learning. J. Neurosci. Methods.

[73] Karl T., Pabst R., von Hörsten S. (2003). Behavioral Phenotyping of Mice in Pharmacological and Toxicological Research. Exp. Toxicol. Pathol..

[74] Bradford M.M. (1976). A Rapid and Sensitive Method for the Quantitation of Microgram Quantities of Protein Utilizing the Principle of Protein-Dye Binding. Anal. Biochem..

[75] Warde-Farley D., Donaldson S.L., Comes O., Zuberi K., Badrawi R., Chao P., Franz M., Grouios C., Kazi F., Lopes C.T. (2010). The GeneMANIA Prediction Server: Biological Network Integration for Gene Prioritization and Predicting Gene Function. Nucleic Acids Res..

[76] Szklarczyk D., Kirsch R., Koutrouli M., Nastou K., Mehryary F., Hachilif R., Gable A.L., Fang T., Doncheva N.T., Pyysalo S. (2023). The STRING Database in 2023: Protein-Protein Association Networks and Functional Enrichment Analyses for Any Sequenced Genome of Interest. Nucleic Acids Res..

[77] Salgado-Hernández S.V., Martínez-Retamoza L., Ocadiz-Delgado R., Pérez-Mora S., Cedeño-Arboleda G.E., Gómez-García M.d.C., Gariglio P., Pérez-Ishiwara D.G. (2025). miRNAs Dysregulated in Human Papillomavirus-Associated Benign Prostatic Lesions and Prostate Cancer. Cancers.

