# Supplementary material for: Aqueous Extract of Bacopa procumbens and the NAPEL Formulation Mitigate MPTP-Induced Neurotoxicity via Nrf2/HSF1/HIF-1α Signaling in a Parkinson’s Disease Model
Source: Int J Mol Sci. 2025 Dec 10;26(24):11914. doi: 10.3390/ijms262411914 (PMC12733197; doi:10.3390/ijms262411914)
Supplement: Supplementary file 1 [file ijms-26-11914-s001.zip › Supplementary tables.pdf]

Supplementary Table S1. Fold change in expression levels of proteins from the Nrf2/ARE pathway in the treatment groups compared to the MPTP group.

	Striatum			Substantia nigra		
Protein	MPTP+L-DOPA	MPTP+Bp	MPTP+NAPEL	MPTP+L-DOPA	MPTP+Bp	MPTP+NAPEL
Nrf2	−1.10	10.43	0.10	−0.30	−1.60	−1.83
p-NRF2	−1.04	5.27	−0.81	−2.96	2.97	−2.43
HO-1	0.67	4.30	−1.80	−0.31	2.26	−0.67
SOD-1	4.83	−0.83	−0.53	0.13	5.50	0.43
CAT	1.00	3.70	−3.23	0.20	6.17	−0.11
GSR	−4.87	2.53	−9.53	2.73	10.33	1.36

Positive values indicate increased protein expression, while negative values indicate decreased expression compared to the MPTP group.

Supplementary Table S2. Fold change in expression levels of HSF1 conformations in the treatment groups compared to the MPTP group.

	Striatum			Substantia nigra		
Protein	MPTP+L-DOPA	MPTP+Bp	MPTP+NAPEL	MPTP+L-DOPA	MPTP+Bp	MPTP+NAPEL
Monomer (55 kDa)	−2.4	5.3	−2.8	0.00	13.50	1.14
Dimer (100 kDa)	−2.03	3.44	−1.86	0.46	7.16	0.36
Trimer (150 kDa)	−0.57	5.60	−0.57			

Positive values indicate increased protein expression, while negative values indicate decreased expression compared to the MPTP group.

Supplementary Table S3. Fold change in expression levels of the lipid peroxidation marker 4-HNE in the treatment groups compared to the MPTP group.

	Striatum			Substantia nigra		
Protein	MPTP+L-DOPA	MPTP+Bp	MPTP+NAPEL	MPTP+L-DOPA	MPTP+Bp	MPTP+NAPEL
4-HNE	0.83	−3.34	−0.10	−0.70	−5.27	−5.14

Positive values indicate increased protein expression, while negative values indicate decreased expression compared to the MPTP group.

Supplementary Table S4. Fold change in expression levels of proteins related to cell signaling and stress adaptation in the treatment groups compared to the MPTP group.

	**Striatum**			**Substantia nigra**		
Protein	MPTP+L-DOPA	MPTP+Bp	MPTP+NAPEL	MPTP+L-DOPA	MPTP+Bp	MPTP+NAPEL
HIF-1α	−1.03	2.00	−1.23	0.10	3.94	−0.66
AKT	−1.27	−0.53	−2.13	−0.30	0.20	−1.04
p-AKT	−0.37	0.53	−0.43	−0.23	−0.23	−0.36
β-catenin	−0.03	0.24	0.30	0.10	0.27	−0.23

Positive values indicate increased protein expression, while negative values indicate decreased expression compared to the MPTP group.
