# Supplementary material for: Aqueous Extract of Bacopa procumbens and the NAPEL Formulation Mitigate MPTP-Induced Neurotoxicity via Nrf2/HSF1/HIF-1α Signaling in a Parkinson’s Disease Model
Source: Int J Mol Sci. 2025 Dec 10;26(24):11914. doi: 10.3390/ijms262411914 (PMC12733197; doi:10.3390/ijms262411914)
Supplement: Supplementary file 1 [file ijms-26-11914-s001.zip › Supplementary Figure S1.pdf]

A

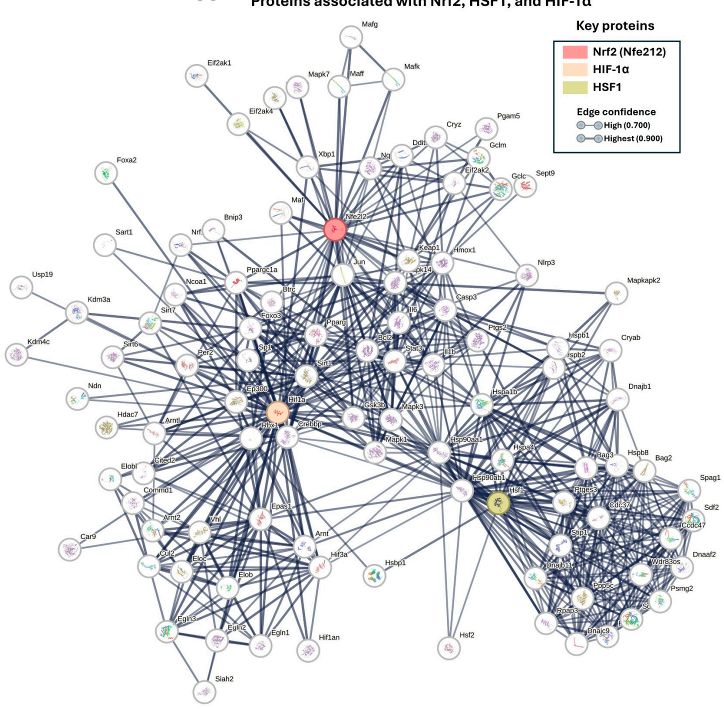

B

Protein	Score	Protein	Score	Protein	Score
Maff	0.977	Hif3a	0.993	Vhl	0.999
Nqo1	0.912	Sept9	0.769	Kdm4c	0.748
Mafk	0.969	Bag2	0.772	Sdf2	0.723
Mafg	0.994	Egln1	0.999	Nrf1	0.733
Hsbp1	0.905	Hspb8	0.859	Hspa4	0.833
Arnt2	0.999	Ep300	0.999	Gclc	0.915
Cited2	0.908	Mapk1	0.948	Ddit3	0.959
Hif1an	0.993	Wdr83os	0.728	Ndn	0.730
Egln2	0.995	Eif2ak3	0.945	Arntl	0.945
Elobl	0.829	Pih1d2	0.724	Arnt	0.999
Sirt1	0.845	Sdf2l1	0.720	Pgam5	0.711
Egln3	0.995	Crebbp	0.999	Psmg2	0.725
Hsf2	0.718	Rbx1	0.967	Gclm	0.829
Dnaaf2	0.723	Mapkapk2	0.819	Eif2ak1	0.921
Hmox1	0.927	Siah2	0.731	Ppargc1a	0.992
		Kdm3a	0.817	Ptgs2	0.826

Protein	Score	Protein	Score	Protein	Score
Sirt6	0.988	Elob	0.994	Casp3	0.778
Hsp90aa1	0.999	Eif2ak4	0.936	Hspb2	0.738
Hspa1b	0.995	Spag1	0.733	Il1b	0.772
Hsp90ab1	0.999	Bnip3	0.771	Dnajb11	0.798
Per2	0.825	Stat3	0.980	Xbp1	0.967
Ncoa1	0.829	Dnajb1	0.971	Il6	0.840
Ccdc47	0.721	Jun	0.870	Eif2ak2	0.954
Maf	0.997	Usp19	0.756	Sp1	0.764
Commd1	0.793	Eloc	0.997	Epas1	0.998
Gsk3b	0.944	Bcl2	0.817	Sart1	0.785
Cryz	0.797	Mapk1[illegible]	0.808	Btrc	0.860
Pparg	0.837	Ppp5c	0.790	Hspb1	0.827
Cdc37	0.871	Stip1	0.855	Cryab	0.735
Car9	0.815	Foxa2	0.910	Nlrp3	0.761
Ptges3	0.897	Mapk7	0.947	Foxo3	0.995
Cul2	0.996	Keap1	0.999		
Hdac7	0.869	Rpap3	0.749		
Mapk3	0.974	Dnajc9	0.756		
Sirt7	0.805	Bag3	0.901		

Supplementary Figure S1. Interactome analysis of the transcription factors Nrf2, HSF1, and HIF-1α with the proteome of *M. musculus*. A total of 100 potential interacting proteins with a probability threshold of interaction equal to or greater than 70% were considered. (A) Protein–protein interaction network showing the connectivity of Nrf2, HSF1, and HIF-1α with their associated proteins, with edge thickness representing different levels of confidence (0.700–0.900). (B) List of the 100 proteins identified as associated, displayed with their respective interaction probability scores.
